# Obituary of Dr. Susumu Seino

**DOI:** 10.1111/jdi.13686

**Published:** 2021-12-13

**Authors:** Nobuya Inagaki

**Affiliations:** ^1^ Department of Diabetes, Endocrinology and Nutrition Kyoto University Graduate School of Medicine Kyoto Japan

I am deeply saddened to report that my esteemed mentor Susumu Seino passed away on April 14, 2021. Susumu left a remarkable scientific legacy to his colleagues, former students, and physicians who benefitted from his many academic accomplishments in diabetes.

Susumu was born in Matsue City, Shimane Prefecture, in 1948, and attended Kobe University as an undergraduate. After graduation, he worked as a physician in the internal medicine department of Kobe University Hospital, Amagasaki Prefectural Hospital and Kitano Hospital for 4 years, during which time he became interested in the field of diabetes through encounters with Drs. Masaki Ikeda and Mikio Yawata.

Susumu began his research at the Department of Internal Medicine, Kyoto University under the guidance of Prof. Hiroo Imura. He studied the regulatory mechanisms of somatostatin, glucagon and insulin secretion, and obtained his Ph.D. in 1982 at Kyoto University. He then studied abroad in the laboratory of Profs. Aaron I. Vinik and Stefan S. Fajans of the University of Michigan. During this period, he joined the laboratory of Dr. Graeme I. Bell at the University of California, San Francisco to learn cutting‐edge techniques of molecular biology. He then joined the laboratory of Prof. Donald F. Steiner of the University of Chicago in 1985. Coincidentally, Graeme was recruited to the University of Chicago, and Susumu and Graeme set up a laboratory and started research on diabetes, incorporating novel molecular biological techniques in earnest. Researchers from all over the world including Japan such as Drs. Hirofumi Fukumoto, Yuichiro Yamada and Jun Takeda gathered at the Chicago laboratory. Susumu instructed them and they succeeded in cloning the insulin receptor gene and clarifying the cDNA of glucose transporters, the somatostatin receptor and calcium channels.

Susumu obtained tenure as an associate professor of the University of Chicago, but was invited to be professor of Chiba University and returned to Japan in 1991. I joined his laboratory immediately after receiving my Ph.D. at Kyoto University in 1992, and set up the laboratory with him. I had the opportunity to do diabetes research using the most advanced molecular biology techniques available at the time under the guidance of Susumu. Fortunately, with the help of electrophysiologist Dr. Tohru Gonoi, we were able to combine molecular biology and electrophysiology, and succeeded in isolating Kir6.2 and determining the structure of β‐cell ATP‐sensitive potassium (K_ATP_) channels as a complex of Kir6.2 and SUR1. It was especially exciting because our results greatly contributed to elucidation of the insulin secretion mechanism on a fuel model in which glucose metabolism is critical for insulin secretion. In fact, many patients with neonatal diabetes or congenital hyperinsulinemic hypoglycemia were found to have mutation of the Kir6.2 or SUR1 genes, and it became clear why sulfonylurea rather than insulin is effective in many cases of these diseases. While Susumu clarified the direction of research, he let the researchers do their own projects in his laboratory. He valued personal motivation, but he also valued the morals of science. On holidays, Susumu often took me to dinner driving his classic Volvo, which he had brought back with him from the United States. The intense period of research with him for more than five and a half years is an invaluable and precious memory for me.

Other researchers and graduate students including Drs. Takashi Miki, Tadao Shibasaki, and Kohtaro Minami joined his laboratory after me, and Susumu moved to his alma mater, Kobe University, in 2003 with them. At Kobe University, Susumu's research developed further by incorporation of analyses such as total internal reflection fluorescence microscopy and metabolomics. He proposed a novel mechanism of insulin secretion in which insulin granules directly fuse to membranes and are released with almost no docking process, differing thereby from the neurotransmitter mode of neurons. cAMP is known to be important as an intracellular signal promoting insulin secretion by incretins in pancreatic β‐cells. Susumu discovered a pathway in this process that is mediated by Epac2. He also found that sulfonylurea binds to Epac2, and proposed interaction of the sulfonylurea and Epac2 signals. Recently, Susumu and his colleagues including Drs. Harumi Takahashi and Norihide Yokoi found that glutamate production is stimulated by glucose and that cAMP facilitates this, establishing that glutamate plays a critical role in incretin‐induced insulin secretion.

Susumu received many prestigious national and international awards including the Hagedorn Award of the Japan Diabetes Society in 2001, the Albert Renold Prize for Outstanding Achievements in Research on the Islets of Langerhans of the European Association for the Study of Diabetes in 2010, The Medal with Purple Ribbon in 2011, the Manpei Suzuki International Prize for Diabetes Research in 2018, and the Japan Academy Prize in 2018. He contributed to the activities of the society as director of the Japan Diabetes Society and the Japan Endocrine Society, and made a great contribution not only as a handling editor of the Journal of Diabetes Investigation but also as the chair of the Award Selection Committee of the Asian Association for the Study of Diabetes.

Susumu loved enthusiastic discussions with any researcher, and was a passionate teacher and mentor of young students. He had a wonderful sense of humor, especially valuing interaction with foreign researchers, recognizing the value of both Japanese and foreign cultures, while striving to harmonize them in a spirit of hospitality. He was a worthy representative of the global Japanese scientist. He left the following message for young people in the award lecture for the Manpei Suzuki International Prize for Diabetes Research entitled “Voyage to the islets of Langerhans: For better understanding and treatment of diabetes”.

Don't stay in one world (society) forever.

Don't be afraid of changing.

Nothing is too late to start.

Treasure your simple and naïve questions and keep pursuing them.

Cultivate your sense as well as logic. (Study hard and play hard!)

Open your heart and enjoy meeting with people.

The diabetes community has lost one of its great leaders. It is an irreplaceable joy to have known Susumu and to have worked with him.

